# Rapid and pragmatic implementation mapping for pre-implementation contextual analysis in multisite implementation trials

**DOI:** 10.3389/frhs.2026.1782980

**Published:** 2026-07-03

**Authors:** Laura Ellen Ashcraft, Morgan A. Watson, Casey Vaughan, Sein Lee, Shimeng Yang, Meghan B. Lane-Fall

**Affiliations:** 1 Division of Epidemiology, Department of Biostatistics, Epidemiology, and Informatics, Perelman School of Medicine, University of Pennsylvania, Philadelphia, PA, United States; 2 Leonard Davis Institute of Health Economics, University of Pennsylvania, Philadelphia, PA, United States; 3 Penn Implementation Science Center, University of Pennsylvania, Philadelphia, PA, United States; 4 Department of Anesthesiology, Vagelos College of Physicians and Surgeons, Columbia University in the City of New York, New York, NY, United States; 5 Department of Anesthesiology and Critical Care, University of Pennsylvania Perelman School of Medicine, Philadelphia, PA, United States; 6 New York-Presbyterian/Columbia University Irving Medical Center, New York, NY, United States

**Keywords:** implementation mapping, implementation research logic model, implementation science, methods, strategy selection

## Abstract

**Background:**

Systematic and data-driven implementation strategy selection is typically a resource-intensive process limited by time and implementation science expertise. Simultaneously, implementation trials often include externally driven timelines. To facilitate the conduct of a multisite hybrid trial, we developed and deployed a tool to facilitate rigorous yet pragmatic implementation mapping for strategy selection.

**Methods:**

As part of the HATRICC-US hybrid type II stepped wedge trial, we developed a strategy selection process based on implementation mapping and the Implementation Research Logic Model. The novel process was refined across two sites, tested in two additional sites, and replicated through the remaining sites of the trial. We considered the resources required, duration, alignment with best practices in implementation science, and usefulness to clinical implementation partners.

**Results:**

The final rapid implementation mapping process included the following seven steps: Step 1—Data Preparation; Step 2—Determinants and Change Objective Identification; Step 3—Summary Table Development & Internal Member Checking; Step 4—Identification of Implementation Strategies; Step 5—Completion of Implementation Research Logic Model; Step 6—Internal & External Member Checking; and Step 7—Completion of Tailored Implementation Blueprint and Manual.

**Conclusions:**

There are often significant resource barriers that prevent the rapid use of implementation mapping and the IRLM and similar tools in implementation settings. We developed rigorous and rapid methods incorporating both implementation mapping and the IRLM for leveraging pre-implementation data collection to inform implementation efforts while also being responsive to both clinical partners and funding deadlines.

**Clinical Trial Registration**: identifier NCT04571749.

## Background

Systematic and explainable implementation strategy selection supports the transferability of implementation research and advances the field of implementation science (1). Implementation science is informed at every phase of the research process (e.g., design, strategy selection, implementation) by the people the innovation is aimed to assist. Implementation strategies are the “stuff” we do to help people, groups, organizations, and communities utilize evidence-based practices (2) with the Expert Recommendations for Implementing Change (ERIC) being the most common implementation strategy taxonomy (3, 4). Common approaches to strategy selection rely on expert opinions, conceptual frameworks, community input, and increasingly, co-design with interested parties. For example, the Consolidated Framework for Implementation Research (CFIR)- ERIC matching tool (an excel macro)^1^ provides high-level guidance based on expert recommendations; however, this tool has yet to be widely rigorously tested.

Systematic selection methods have emerged, such as concept mapping (5), which creates a visual map connecting concepts and specific dimensions, and intervention mapping, which incorporates theory, evidence, and perspectives from invested parties to develop strategies (6). Implementation mapping is another common approach to support the identification, selection, and specification of factors needed for implementation (7). Implementation mapping builds on intervention mapping by expanding the fifth step of planning for real-life adoption, implementation, and sustainability by identifying users and their needs (7). The five tasks of implementation mapping are as follows (7): 1. Conduct a needs and assets assessment; Identify adopters and implementers; 2. Identify adoption and implementation outcomes, performance objectives, and determinants. Create change matrices; 3. Choose theoretical methods and select/create implementation strategies; 4. Produce implementation protocols and materials; 5. Evaluate implementation outcomes.

The implementation research logic model (IRLM) was built, in part, on the implementation mapping process and creates a way to guide the planning and execution of an implementation effort by specifying determinants, strategies, and outcomes (8). These tools can be used across theories, models, and frameworks [e.g., CFIR, Health Equity Implementation Framework (9)] which allows for applicability across a broad set of contexts, topics, and innovations.

While the goal of implementation mapping has been to more clearly specify how an implementation strategy is selected (10), there may still be missing pieces in how it is applied. This may include the identification of mechanisms and how the components in each task build connections to one another. Further, the implementation mapping process itself is relatively ambiguous in that it allows for significant variation in how the five tasks are conducted (7, 10). When used in conjunction, implementation mapping and the IRLM can guide efforts in implementation strategy selection and integrate clinical partners and other shareholders (11). This potential increase in efficiency and transparency has limitations in that they take significant time and resources.

In a hybrid type-II cluster randomized stepped wedge trial, we sought to leverage the power of implementation mapping and the IRLM by developing a rapid analysis approach responsive to the needs of our clinical partners and building on the existing body of literature.

### Study objective

Our objective was to develop and test a rigorous and pragmatic approach to leverage the power of implementation mapping and the IRLM in the pre-implementation or contextual inquiry phase of an implementation trial. We define pragmatic as a process that is responsive to the needs of clinical partners in its time efficiencies, is adaptable given organizational structures, and can be conducted given available resources. Throughout the description of our process, we identify resources used, duration, and potential adaptations for resource-limited settings. We sought to 1) be responsive to the needs of our clinical partners and 2) track across determinants, change objectives, implementation strategies, outcomes, and to identify potential mechanisms and causal pathways related to implementation success.

## Methods

### Context: HATRICC-US

**Figure 1:**
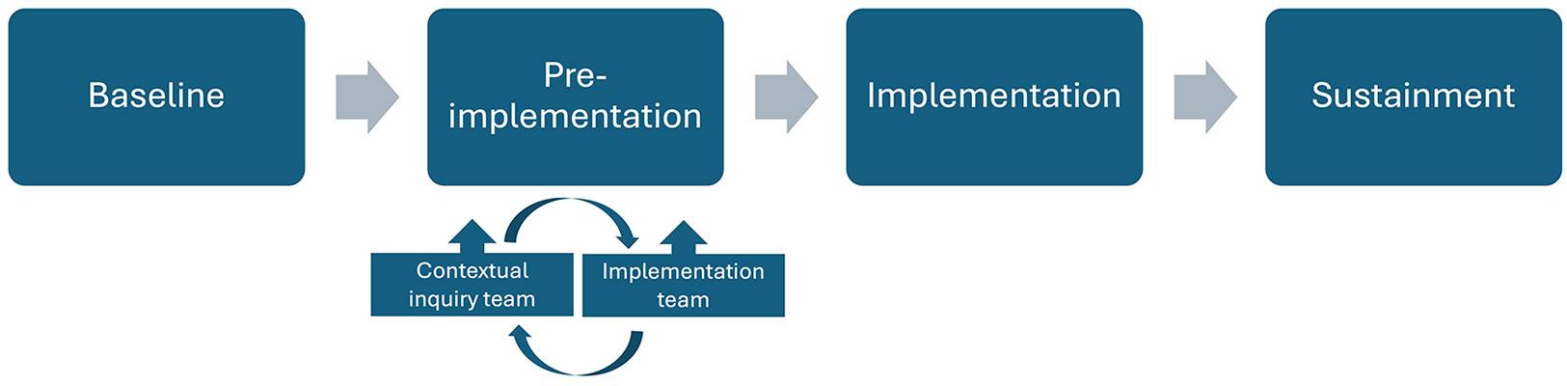
Study flow for each HATRICC-US site (OR-ICU pairing) with timing of contextual inquiry and implementation mapping process.

Handoffs and Transitions in Critical Care—Understanding Scalability (HATRICC-US) is a stepped-wedge cluster randomized hybrid type II implementation-effectiveness trial testing 1) the effectiveness of a standardized handoff between the operating room (OR) and intensive care unit (ICU) and 2) universal and tailored implementation strategy bundles in 12 ICUs (ten adult and two pediatric) (12). For the purposes of this study, a site was defined as an operating room-intensive care unit pairing. Site participation in HATRICC-US consisted of four phases, including baseline, pre-implementation (i.e., contextual inquiry), implementation, and sustainment (see Figure 1).

The evidence-practice gap is structured communication between the OR and ICU upon patient transfer to the ICU, which when absent may lead to preventable patient harm. The innovation of interest is a standardized handoff process which includes a ten-step process whereby the OR and ICU teams gather post-patient stabilization in the ICU and communicate patient status and treatment information. As an implementation-effectiveness hybrid type II trial, HATRICC-US focuses on both the effectiveness of the innovation (i.e., standardized handoff) on patient outcomes and testing implementation strategies that include both universal/shared strategies (e.g., education, champion, and creation of an implementation blueprint) and site-specific tailored implementation strategies.

The current manuscript focuses on the process used by the HATRICC-US team to use pre-implementation and contextual inquiry data to inform the selection of implementation strategies for each ICU of the first six ICUs enrolled in the HATRICC-US trial. The six ICUs were all adult ICUs and included medical/surgical (*n* = 1), trauma/mixed surgical (*n* = 3), neuro (*n* = 1), and cardiovascular (*n* = 1).

**Data collection**. During the pre-implementation phase, the contextual inquiry team conducted virtual and/or in-person site visits with each site depending on COVID-19 visitation restrictions at the time pre-implementation was scheduled to start. The contextual inquiry team was led by a senior systems and human factors engineer and included a post-doctoral trainee and staff analyst. During these site visits, the team conducted a series of semi-structured interviews and process map exercises focusing on the current process of the transfer of a patient from the OR to ICU including any existing handoff activities.

The implementation team was led by the study PI (Initials: MLF) and an implementation science methodologist (LEA) and included a project manager (CV) and research assistants (MAW, SL, and SY). The implementation team analyzed qualitative data (i.e., interviews, focus groups, in-person observations, open-ended text from surveys) collected by the contextual inquiry team to inform the development of tailored implementation strategy bundles (see Figure 1). This process is described in full in Steps 1 and 2 below. The implementation team conducted domain level coding to all seven domains of the Tailored Implementation for Chronic Diseases' (TICD) checklist (13).

### Impetus for a novel process

The process was initially developed out of necessity to meet the needs of our project. The study PI (MLF) identified the use of implementation mapping as a critical approach to understanding the ways in which determinants can be mapped to change objectives, implementation strategies, and finally, outcomes. However, in the initial implementation analysis process, we identified that traditional approaches required extensive resources and were not responsive to the needs of our clinical partners in that they took too long. Our desire to meet the needs of our clinical partners and leverage existing best practices was the stimulus for the development of the novel implementation mapping process. We then completed within-team user testing and refinement with two additional sites before replicating the process with trained IS analysts at another two sites for the remaining four sites.

### Development of process

The initial process developed organically in that the two implementation scientists (MLF and LEA) had a general sense of what the end products for the site could look like (see Results, Step 7) and approximated how to get there, but the stepwise fashion had not yet developed. We used the first HATRICC-US site to conduct the initial development of a process by writing down each step we completed throughout the implementation analysis (including implementation mapping and the IRLM).

We found that solidifying key definitions based on the existing literature was critical to ensuring consistency and successful replication of the process. These definitions include:

Implementation determinants: Historically called barriers and facilitators, these are the individual, group, organization, and/or community factors that influence the ability to put the innovation into use. Determinant frameworks such as the TICD Checklist (13) and the Consolidated Framework for Implementation Research provide a list of potential implementation determinants (14, 15).
Change objectives: Discrete actions taken by implementers to reach a specific goal or objective connected to an implementation determinant (7).
Implementation strategies: All the things we do to help individuals, groups, organizations, and communities to utilize and sustain the practice of interest [based on the language proposed by Curran (2)]. The Expert Recommendations for Implementing Change provides a taxonomy of implementation strategies (3).
Socioecological levels: The different groupings of people at which we intervene. This often includes the individual, group, organization, and society [based on Bronfenbrenner's Ecological Systems Theory (16)].
Implementation phases: The process each site experiences between the initial implementation of the standardized handoff to the point of sustainment. This includes early implementation and training, through transitioning the innovation into routinized care.

### Initial testing with expert use and refinement

The two implementation scientists tested and refined the process for the second and third HATRICC-US sites, two sites total. For the second and third sites, a PhD-trained implementation scientist (LEA) completed each step of the process (as outlined below) and documented additional details for each step including adding estimates of how long each step may take. For the third site, the implementation scientist (LEA) developed a training protocol to enable the process to be completed by analysts. The training primarily focused on the theme and determinant identification (see Results, Step 2).

During the initial testing and development, we solicited feedback from our clinical partners. For the first site, we shared the entire IRLM and full implementation blueprint (see Results, Step 7) which provided extraneous details that clinical partners found confusing. For the second site, we prioritized sharing the themes we found from the mixed methods data collection and described the process to identify how to move forward based on the evaluation of the current state. We then described how the proposed implementation strategies in the IRLM related back to the implementation determinants identified during the generation of the IRLM (see Results, Step 2). This process was better received and appreciated by our clinical partners.

### Analyst implementation

The training protocol was implemented for two entry-level implementation analysts using data from site three. These two analysts then completed the implementation mapping process for the fourth site and one analyst completed the process for the fifth site. The analysts' evaluation was supervised by the project manager (CV) and implementation scientist (LEA). This supervision ensured adherence to the evaluation protocol and was responsive to questions and potential changes needed to the process. For example, we found that clinical partners were less interested in the embedded details of the completed IRLM (as was done with the first site) but instead wanted to hear high-level themes identified in data collection and the operationalization of the recommended strategies. By site five, no additional changes were required to the implementation mapping protocol.

## Results

The final rapid implementation mapping process included the following seven steps. At the time of publication, the following process has been used with six OR-ICU pairings. Across the six sites, there was an average of 21 determinants (range: 16–25); 12 change objectives on average (range: 11–17), and an average of 6 implementation strategies (range: 4–8) across the implementation and sustainment phases (see Table 1).

**Table 1:** Description of six participating OR-ICUs pairs.

HATRICC-US site number	Number of determinants	Number of change objectives	Number of strategies
Site 1	25	17	5
Site 2	23	15	6
Site 3	21	11	4
Site 4	22	11	4
Site 5	21	11	7
Site 6	16	12	8

**Figure 2:**
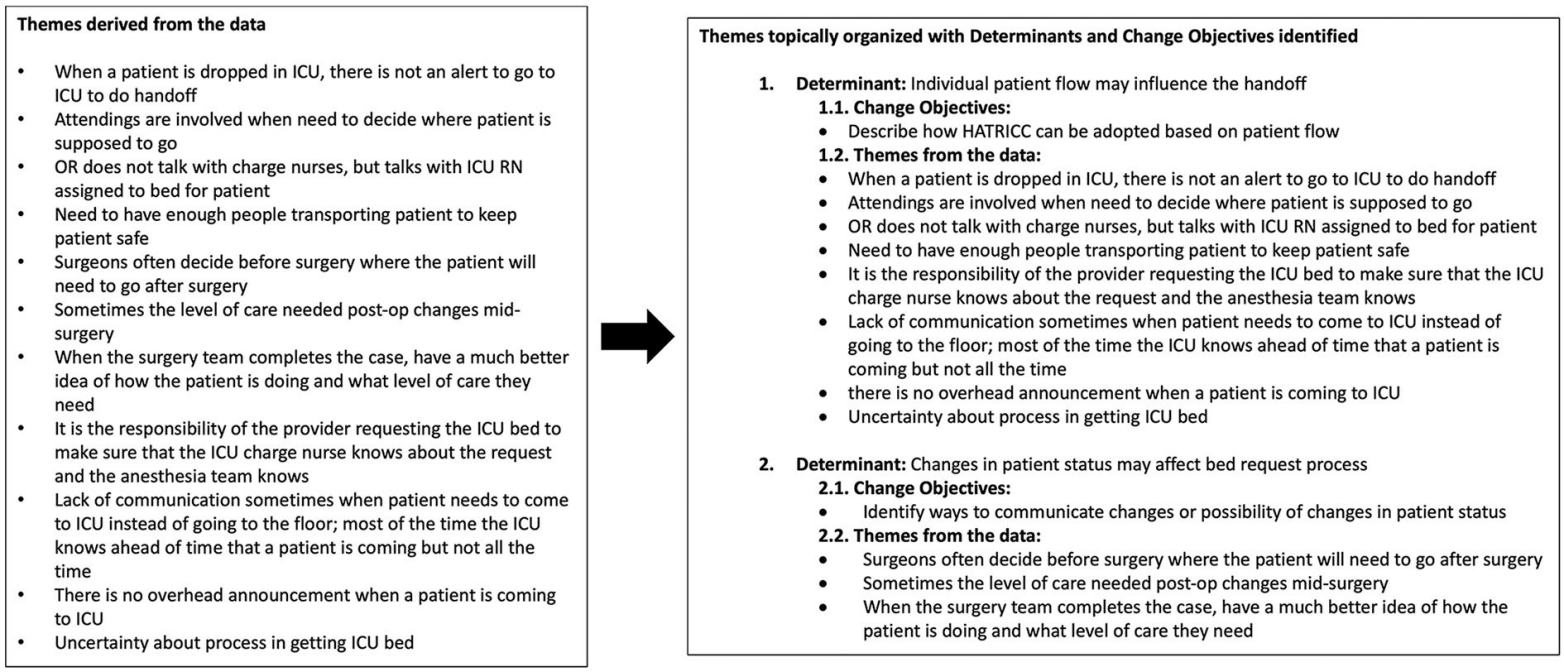
Example of theme identification and organization into determinant-groupings with change objectives.

**Figure 3:**
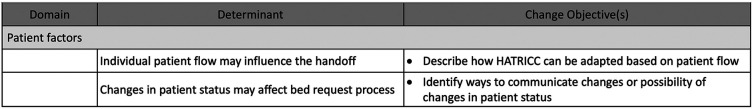
Example of portion of summary table after internal member checking.

**Figure 4:**
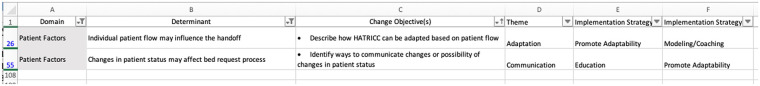
Screenshot of implementation strategy identification process spreadsheet.

**Figure 5:**
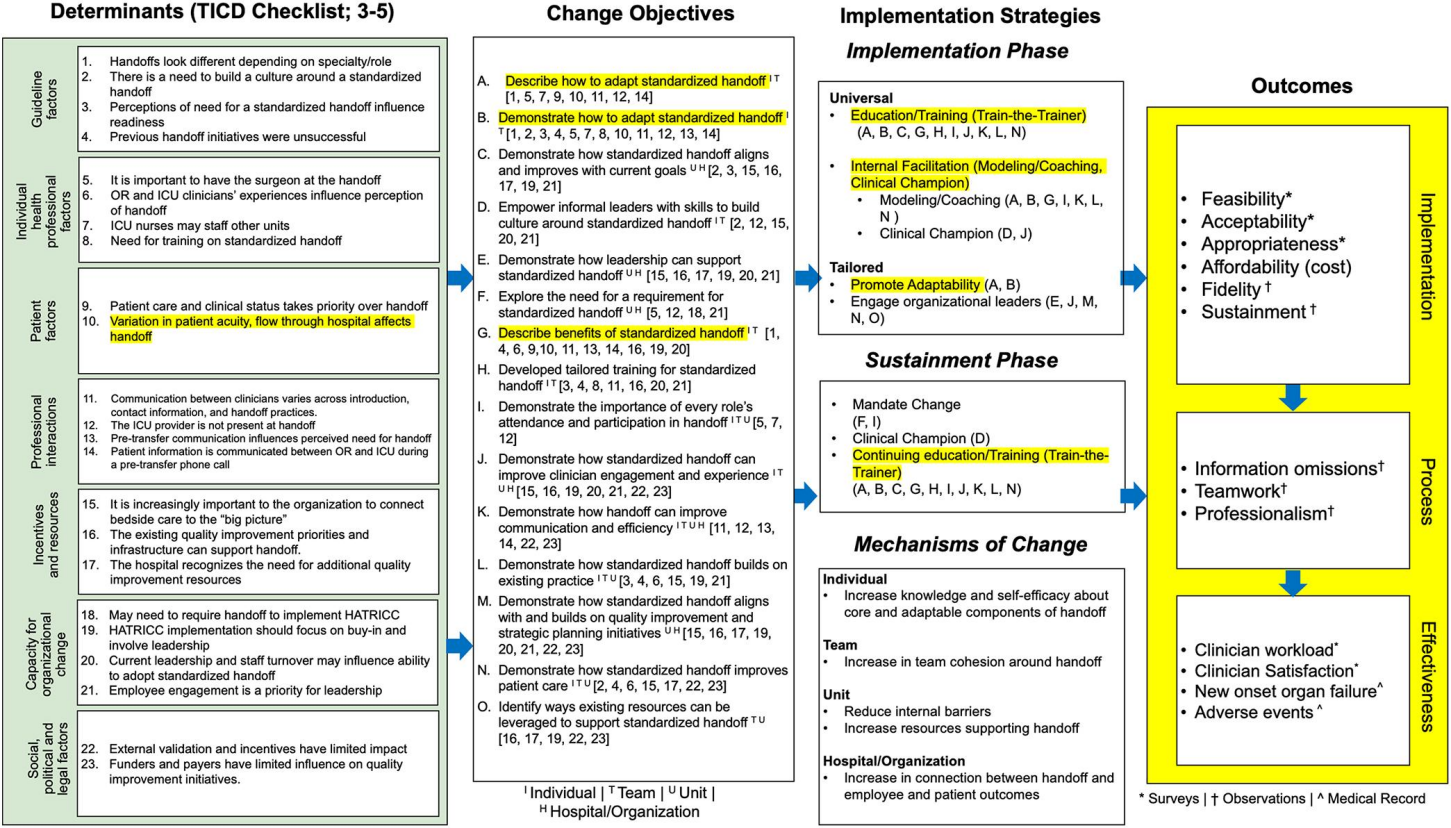
Example of implementation research logic model with example determinats, change objectives, and implementation strategies highlighted.

**Figure 6:**
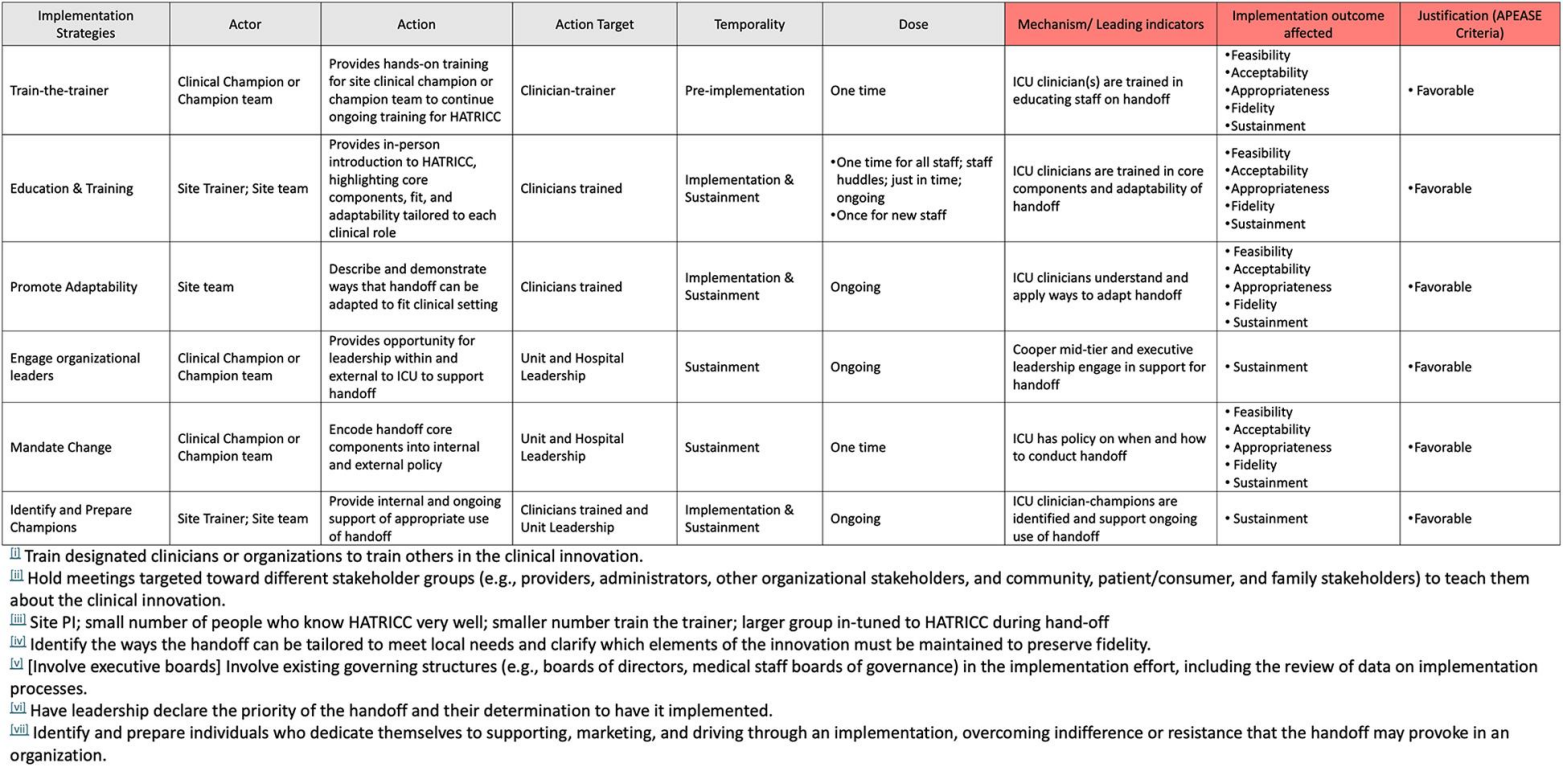
Example implementation blueprint for internal use.

The following describes the final process we used for each site. In each step, we provided examples from the HATRICC-US trial in Figures 2–6. The examples provided are real and the content should be considered in progress and preliminary. We provided these examples to support transparency and future hypothesis generation. Templates of all documents can be found in Supplementary File 1.

### Analyst training

The lead implementation scientist (LEA) led three rounds of training for this process. Across the three rounds, analyst trainees included one project manager, two masters students, one bachelors-level research coordinator, and one doctoral student. Trainee analysts had various degrees of experience with implementation science and qualitative data collection and analysis.

Training began with an overview of the seven steps and goals for the analysis. The stated goal was to use data from the contextual inquiry team to first identify factors that influence the ability for the site to implement the standardized OR to ICU handoff (implementation determinants) and then to identify approaches for that site to use their existing strengths to overcome any barriers (implementation strategies). This training step was a one-time 60–90 minute meeting.

Next, training focused on the identification of themes from the data (see Step 2 below). Analyst trainees used data from Site 1 to practice identifying implementation-related themes. The number of rounds of theme identification ranged from two to three and depended on how quickly analysts were able to demonstrate consistency in identifying themes and the degree of agreement between analyst responses. This training step took between two and three weeks. Training for implementation determinant and change objective identification involved two to three rounds of practice with iterative feedback. Trainee analysts worked together to resolve conflicts and met with the lead implementation scientist to discuss questions.

Training for the remaining steps was experiential in that due to external project deadlines, analysts worked alongside the implementation scientist to create and edit the summary table, select implementation strategies, and generate the IRLM and implementation blueprint. This learning occurred both asynchronously via feedback on drafts or synchronously via screen sharing and verbal processing through the implementation strategy selection process.

In total, training took approximately 5–6 weeks with some training rounds taking longer due to scheduling conflicts and trainee analyst roles on other research projects.

### Step 1. Data preparation

#### Data sources

We recommend following best practices to conduct contextual inquiry, including using a determinant framework to guide data collection and collecting data that reflect multiple levels of the social ecological model. Our data sources included artifacts and notes from site visits; transcripts from interviews and focus groups; summary data from pre-implementation surveys; and text from open text fields on observation tools developed for this study. We opted to collect data in this manner to strategically capture TICD constructs of interest while respecting clinicians' limited availability to participate in research. For the mapping process, the most useful data were drawn from pre-implementation contextual inquiry qualitative data collection. This included process map interviews, interviews with site-level senior leaders (e.g., hospital chief executive officer), site PI interviews, a member checking meeting with OR and ICU providers, and open-ended survey data from surveys administered at the site. The average length of every qualitative piece of data collection (all interviews and focus groups) was 47 minutes. The average length of the member checking and design session meetings was 58 minutes. The average of process map interviews is 49 minutes. Site-level leadership (hospital CEO or CMO) interviews were 33 minutes on average. The data generated through these data collection methods were used throughout the rest of the implementation mapping process.

#### Domain-level coding

Next, all qualitative data were coded using a rapid and deductive thematic qualitative analysis process. All codes for the qualitative data were at the domain level or “higher level” of the determinant framework used and conducted in NVivo. During this coding process, we avoided assigning two codes to the same text as we found the nuances in the data were still identified in Step 2, even when domains overlapped. We used the TICD checklist determinant framework to guide our data collection and analysis. One research coordinator coded all qualitative data from each site into the seven TICD checklist domains.

#### Step 1 timing, supervision, and resource requirements

After data collection, the Domain level coding included a trained undergraduate student using NVivo and took about 6–8 h to code 5–6 transcripts. The student analyst was supervised by the master's trained project manager (CV).

### Step 2. Determinants and Change Objective Identification

The second step was to identify determinants and change objectives. Step 2 was the most labor intensive of the seven steps, and a team of the data analysts was formed based on the quantity of the data to be analyzed. Each analyst was onboarded and understood the specific metric that the analysis should focus on, along with specific implementation factors rather than every meaning (as is the case with qualitative analysis).

During this step, implementation analysts reviewed each set of data under each TICD Checklist domain and generated a list of nonevaluative and concise theme statements representative of the data. Some examples include: “verbal handoff happening at bedside” (site 3) and “handoff sheets are color-coded by specialty” (site 6; see Figure 2 for more examples from our data). The goal of this process was to identify themes focused on factors which may impact implementation identified throughout the data.

For example, for a quote from the Professional Interactions domain from Site 06, “*…we’ll try to do it face-to-face… The goal is to have it face-to-face so that you know that the message was given and received, that's not what always happens.*” This quote was summarized as, “clinicians prefer to communicate about OR-to-ICU transfers in-person rather than Epic chat.” In this way, the underlying theme of the data is maintained without positive or negative valence.

Next, we grouped similar themes (see Figure 2). For example, all theme statements that described the importance of prioritizing patient care were grouped together. From each grouping, analysts developed a determinant statement for each summarized category. This process was repeated across each domain coded in the data. Upon completion of theme identification for all domains, the determinants were reviewed for alignment with that domain and for duplication. Determinants and their corresponding theme statements were moved to the best fitting domain and duplicate determinants and themes were combined.

On the rare occasion a determinant fit into multiple domains, we listed them in both places and annotated this duplication. Finally, for each determinant, the analysts generated change objectives (see Figure 2) which helped to connect implementation determinants with strategies. Throughout this process, the implementation team met weekly for 30 minutes to 1 hour to discuss any questions and discrepancies. All disagreements were resolved by consensus with the senior implementation scientist (LEA) making the final decision.

#### Step 2 timing, supervision, and resource requirements

Step 2 was the most time and resource intensive. All analyses were conducted in Microsoft Word and Excel and were completed by bachelor's and master's trained analysts. Depending on the number of data sources, Step 2 took approximately 15–20 hours to complete in total. The analysts were supervised by a senior implementation scientist (LEA) which occurred weekly. Depending on the site, it took approximately 4–6 weeks to complete Step 2.

### Step 3. Summary table development & internal member checking

The determinants and change objectives were entered into a Summary Table document (see Figure 3). The Summary Table had separate sections for each domain with sub-columns for determinant, change objectives, and cross-listing in multiple domains. This table was reviewed in detail by the implementation scientists and analysts. This internal member checking reviewed the domains, determinants, and change objectives and took between 30 minutes and 1 hour to complete. The group reviewed, edited, and reconciled language, potential duplication, and differences of determinants. The results were a finalized list of determinants and change objectives in each domain.

#### Step 3 timing, supervision, and resource requirements

Step 3 took approximately 2 hours of an analyst time to construct the Summary Table and a single 30–60 minutes meeting for internal member checking. For the earlier sites, the Summary Table was completed by the senior implementation scientist (LEA) and in later sites, it was completed by analysts (SL). All Summary Tables were completed in Microsoft Word. The senior implementation scientist (LEA) provided supervision during Summary Table development and during the internal member checking meeting.

### Step 4. Identification of Implementation Strategies

The final summary table was copied into a spreadsheet. Each change objective received one line (see Figure 4). The corresponding domains and determinants were copied across each connected change objective. The spreadsheet was sorted by change objective to begin to identify common change objectives across domains and determinants. These change objectives were grouped into theoretical themes by the PhD-trained implementation scientist (LEA). The theoretical themes included relative advantage, adaptations, core components, relative fit, etc. The process continued by sorting each column, theme and change objective, until each change objective had a proposed implementation strategy.

Finally, after reviewing the determinants, change objectives, and theoretical groupings, proposed primary and secondary implementation strategies were identified. These strategies were primarily based on a set of “universal” implementation strategies based on the experience with the previous HATRICC trials. We anticipated that a core set of implementation strategies would be required to implement the standardized handoff (e.g., education, champion, and creation of an implementation blueprint). If none of the universal implementation strategies alone met the identified needs, we reviewed the ERIC implementation strategy taxonomy for strategies.

In all six ICUs, the “universal” strategies identified *a priori* were necessary but insufficient to address the identified implementation determinants. For example, one site required internal policy changes to ensure sustainability of the standardized handoff. Other site required additional resource sharing and engaging organizational leaders. While there were some differences across sites, the variation was limited as all sites were adult ICUs.

#### Step 4 timing, supervision, and resource requirements

Step 4 took approximately 4 hours to completed and was completed in Microsoft Excel. The senior implementation scientist (LEA) completed Step 4 for the first four sites and the remaining two sites were completed by an implementation analyst (SL). Supervision for analyst completion was done by the senior implementation scientist (LEA) during weekly 30-minute team meetings.

### Step 5. Completion of implementation research logic model (IRLM)

The findings from Steps 3 and 4 were entered into an adapted Implementation Research Logic Model (IRLM). To make our version of the IRLM, we inserted a change objective section to the right of the determinants. This follows the implementation mapping process where change objectives are used as an intermediary between implementation determinants and implementation strategies (7). The other change to the original IRLM included differentiating implementation strategies between those used in the initial implementation phase and those used in the sustainment phase of the implementation of the innovation.

The determinants section had separate sub-sections for each domain in the theoretical framework. We reviewed the summary table generated in Step 3 and selected 3–5 determinants from each domain that most fully encapsulated determinants from that domain. Each determinant was numbered continually across all theoretical domains. The corresponding change objectives were listed in the next column (moving from left to right) enumerated using letters. After each change objective, we listed the number(s) of the corresponding determinants as well as superscripts indicating at what level the change objective would occur (i.e., individual, team, unit, or hospital/organization).

We next listed the proposed implementation strategies separated by strategies for the initial implementation phase and those for the sustainment or maintenance phase. We used the spreadsheet generated in Step 4 to list the corresponding change objectives after each implementation strategy. Below the implementation strategies, we identified our hypothesized mechanisms of change at the individual, team, unit, and hospital organization levels. Identifying and testing mechanisms of change was not the primary focus of our project. However, we wanted to document potential mechanisms through which change was occurring for transparency and future hypothesis generation.

Finally, we listed our implementation, process, and effectiveness outcomes with subscripts indicating how each outcome is collected (i.e., survey, observations, and/or the electronic health record). The completed implementation research logic model displays the identified determinants, change objectives, implementation strategies, mechanisms of change, and outcomes and how they're connected for each implementation instance using numeric and alphanumeric labeling (see Figure 5).

#### Step 5 timing, supervision, and resource requirements

Step 5 took approximately 5–6 hours with the most time consumer part being the linkages of items across the fields within the IRLM. The initial drafting of the IRLM was completed by the senior implementation scientist (LEA) with later versions completed, with supervision, by the analyst (SL). We used Microsoft PowerPoint to generate our IRLM.

### Step 6. Internal & External Member Checking

The finalized IRLM was shared in an internal member checking meeting between the contextual inquiry and implementation teams. In this meeting we reviewed determinants, change objectives, and implementation strategies, specifically with the data collection team. The goal of this discussion was to identify gaps in understanding between those who conducted the data collection (i.e., the contextual inquiry team) and team members who conducted the implementation analysis process. This discussion often anchored in key factors of importance that arose during the site visits that should be included in the implementation plan. For example, one site had an existing hand-off process that was internally developed by a deceased member of the clinical team. The contextual inquiry team ensured that any implementation plan proposals included the existing hand-off process, in part to respect the contributions of the prior team member.

Next, we shared the results of our implementation mapping process with our clinical partners at the site. We communicated implementation determinants and the recommended implementation strategies. Clinical partners (often unit leadership including the site PI) were asked to identify gaps in the study team's understanding of the current state. In addition to receiving feedback on the IRLM, the goal was to present a list of proposed solutions and not just a list of determinants or barriers via the change objectives and mechanisms of change.

After both meetings, we incorporated feedback from both the study team and clinical partners and generated a final implementation research logic model. For example, clinicians at a site expressed confusion about the procedure for patient flow between the operating room, step-down unit, and intensive care unit. In our initial analysis, this was listed as a significant determinant of the handoff process. However, in the member checking process, we learned that since our time of data collection, additional procedures had been developed and standardized at the site and this was no longer a source of confusion.

Member checking sessions were completed at the convenience of the implementing clinicians. This ranged from a single 30-minutes check-in meeting to asynchronous feedback via email. Our goal was to provide resources and support in a way that met the implementers needs without adding burden.

#### Step 6 timing, supervision, and resource requirements

Outside of the member checking meetings themselves, preparation for the meetings required between 30 minutes and 1 hour by the implementation team, primarily the implementation analyst. All report outs were conducted virtually using Microsoft Teams or Zoom.

### Step 7. Completion of tailored implementation blueprint and manual

The final step in the process involved taking the results from the implementation analysis completed in Steps 1–6 and packaging them in a way that would be most useful to our clinical partners and front-line implementers, described in detail below. We generated several documents to support the implementation of the standardized handoff in each ICU.

#### Implementation blueprint

We created an implementation blueprint or table of each proposed implementation strategy, operationalized using the Proctor criteria (see Figure 6). The goal of the implementation blueprint was to have a one-page document for the frontline implementation team. We named the strategy, identified actors (i.e., those carrying out the strategies), what the strategy would look like in practice (Action), and the action target. For each implementation strategy, we included the original definition from the ERIC taxonomy in a footnote below the table. We included the temporality of the strategy (i.e., pre-implementation, implementation, or sustainment) and the dose (or how frequently the strategy would occur).

For internal study team purposes only, we included columns for the mechanisms, leading indicators, implementation outcomes, and a review of the acceptability, practicality, effectiveness, affordability, safety, and equity (APEASE) criteria (17). This document was internally reviewed by the study team for gaps and points of potential confusion before the finalized document was generated. In this way, we provided the version of the implementation blueprint focused on the information of most use to our clinical partners.

#### Implementation guide

We generated an implementation guide or handbook for each implementing unit with tailored step by step instructions for implementing the standardized handoff. The implementation guide used hospital-specific branding and language (e.g., naming the standardized handoff HATRICC or another hospital-specific name). The implementation guide included a brief background on the need for the standardized handoff, the core components and goals of the standardized handoff itself, and a summary of the data collection, analysis, and findings of the contextual inquiry process, including a copy of the implementation blueprint (excluding the last three columns in salmon headers in Figure 6).

#### Step 7 timing, supervision, and resource requirements

Step 7 required two steps, the development of the implementation blueprint and the tailoring of the implementation guide. The blueprint was completed in 30 minutes to 1 hour by the senior implementation scientist. The implementation guide template was developed for Site 01 and tailored to meet the needs and branding for Sites 02–06. After the initial development, the tailoring took between 1 and 2 hours and was sent to the site PI for review prior to finalization by the project manager (CV). Step 7 was completed in Microsoft Word. The senior implementation scientist and PI provided supervision asynchronously for Step 7.

### Duration and adaptations

Overall, the HATRICC-US implementation mapping process took between six to eight weeks for each site. This process could have been completed in a shorter period; however, sites often had competing initiatives and scheduling challenges which gave us more time to complete our implementation mapping process. We adapted our process based on the number of data sources (i.e., transcripts and surveys) and on timelines as guided by the sites. Throughout the process, a trained bachelor's or master's level analyst could complete most of the activities. There is a requirement for someone with more implementation science knowledge to provide guidance and supervision, specifically around the selection of implementation strategies.

### Comparison across sites

Our process was flexible to the needs of each site from data collection through our rapid implementation mapping process. For example, we solicited timelines and deadlines for each site from site ICU-level leadership. As previously described (see Step 4), we tailored the recommended implementation strategies to the needs of the ICU. We found that the tailored implementation strategies for each site varied depending on the identified implementation determinants.

## Discussion

Pre-implementation and contextual inquiry often fall short of meeting the needs of clinical partners. Existing methods, including implementation mapping and the IRLM, require significant resources and can take several months to complete. This approach is not responsive to the needs of clinical or community partners. We developed a seven-step approach which leverages the power of the implementation mapping process and IRLM tool and can be completed in a shorter period of time. We use the scaffolding of implementation mapping and the organization of IRLM to assess the current clinical context and collaboratively provide implementation recommendations. This process rapidly synthesizes data about the innovation context and connects implementation components (e.g., determinants, change objectives, strategies) with the social-ecological level and timing (pre-implementation, implementation, and sustainment).

The integration of implementation mapping and IRLM allows for improved efficiency of the implementation mapping process while ensuring that the selection strategy is informed by frameworks. Our work builds on existing selection approaches which may result in broad and inconsistent strategy selection or relying on implementation experts who may not provide clear explanations or motivations for their strategy choices (18). Our approach of integrating the IRLM adapts traditional implementation mapping by creating a structured strategy selection method which is replicable and transparent. The IRLM requires researchers to clearly define each step of the strategy selection process which allows for shorter analysis time and easily identifiable areas for adjustments to support scientists assessing interventions and the organization sustaining them. Further, this approach strengthens other systematic approaches (e.g., intervention mapping) which have been criticized for requiring specific training and methodological consultations by developing a clear, easy-to-follow process with explicit frameworks and deliverables that allow for rapid dissemination and iteration (1).

Previous uses of the IRLM have identified proposed implementation mechanisms (19–24), few have attempted to specifically connect the determinants to implementation strategies (19, 20, 23). We built on this work by specifically linking determinants with change objectives and change objectives with implementation strategies. These links can be visualized respectively by the bracketed enumeration following determinants and lettering following change objectives in Figure 5. We specified the ecological level at which the implementation strategies and change objectives occur and at what point during the implementation process. This moves contextual inquiry implementation analysis toward transparency. This contribution specifies and connects the current and future states and highlights the *how* of the implementation process.

The identification and specification of mechanisms are increasingly important to understand the *how* of a given implementation instance (25–27). An early critique of the IRLM is that it did not go far enough to identify the links between determinants, strategies, and outcomes to identify the mechanisms of behavior change (28). To date, several examples of the IRLM attempt to address this gap by including the identification of potential or hypothesized implementation mechanisms (19–24).

The amount of transparency provided by researchers about the implementation process varies widely. Guidelines for transparency have been developed for specific aspects of implementation, but not the entire process. For instance, Proctor et al. highlighted the lack of consistency in the usage of implementation strategies and produced guidelines for the naming, defining, and operationalizing of implementation strategies (29). Without the IRLM to assist in developing a clear way to present the connection between all steps of the implementation process, researchers must rely on each other to clearly document and explain their methods. Garbutt et al. provided a prime example of transparency with their detailed explanation of their use of the CFIR to inform their utilization of the Behavior Change Wheel and the Theoretical Domains Framework in identifying determinants and strategies for implementation (30). The IRLM improves the ease by which researchers can achieve transparency.

External deadlines often drive the implementation evaluations in which these tools are used. Funding and/or reporting deadlines require investigators to prioritize the funders' goals over those of implementer and community collaborations. This may prompt investigators to complete the implementation mapping and IRLM processes without implementer feedback. Previous work has addressed this gap by operationalizing how to evolve and engage community and clinical partners in the implementation mapping process (31). However, even with these advances, implementation mapping and IRLM development still require significant expertise, time, and resources.

The current finalized rapid implementation mapping process was developed within a resource-rich academic and clinical environment in its application within the HATRICC-US study. As such, individuals or organizations in community-based or under-resourced settings may not be able to immediately apply the process as currently written; however, adaptations can be made to adjust the process for various settings. A minimum of two team members is required to undergo analysis and conduct internal checks. To limit potential challenges regarding qualitative data collection, researchers can determine one form of data (e.g., focus groups) instead of the multiple utilized in the HATRICC-US study.

We recognize the need to balance the demands of funders, the needs of clinical partners, and the desire by scholars to conduct rigorous evaluation. This work developed out of our own need to meet these needs within resource constraints. Moving forward, evaluation teams may further refine this process with the goals to increase the resources required and be more responsive to the needs of clinical partners. Additionally, future work may also consider the quantification or strength of the relationships between factors across the IRLM, such as using valence rating (23, 32).

The process developed for this study represents a systematic, theory-driven approach to implementation strategy selection that is explainable to research staff and clinical partners and which acknowledges the importance of selecting strategies with an actual or putative mechanistic link to contextual determinants. Our process differs from implementation mapping as described by Fernandez et al. (7) in at least two ways. First, we do not start with ensuring “that all adopters and implementers important to implementation have been identified” before other mapping tasks can be undertaken (7). Of course, site investigators and local champions are part of the data collection process, but we started with the assumption that additional important implementers would be identified as change objectives emerged and implementation strategies were selected. Our experience has borne this out; some strategies require institutional engagement outside the local setting that would not have been identified from the outset. Examples of this engagement include information technology professionals and local leaders outside the ICU. Involving such individuals at the outset of the needs assessment process could endanger the researcher-participant relationship if research involvement is seen as wasting the professionals' time. Second, we do not assign determinants or implementation outcomes to individual actors; the process of interest in this study is by definition a team process, and individual actors have limited agency to change the process or context in isolation. Rather, we consider determinants by domain and assign actors only at the change objective stage. Our process differs from the IRLM in that we explicitly link specific determinants to specific change objectives and specific change objectives to corresponding strategies (i.e., we “show our work”). In this way, the working assumptions of the team are evident and can be challenged or reaffirmed by stakeholders or other researchers.

### Limitations

There are several limitations to this study. First, the development of the rapid implementation mapping and IRLM process all occurred within a single high-resource setting including significant study-level and institution-level support and implementation science knowledge. We did not explicitly test our process in a lower resource or settings with low implementation science knowledge. We attempted to address this by conducting several rounds of the process relying more on study analysts and by providing all process templates in the Supplementary Files. We recognize that even using this approach contexts without implementation science or implementation practice knowledge may struggle to complete the rapid implementation mapping process. This provides an opportunity for collaborations between high and low resource settings in a way that is not extractive but instead leverages existing resources to support implementation.

Second, this process was developed and tested in a singular type of clinical intervention and setting. Replication of this process in another setting may experience different results. For example, in lower resource settings, not having personnel for coding or analysis may make this process more challenging.

## Conclusion

The implementation mapping process and implementation research logic model tool are both powerful ways to leverage information gathered during the contextual inquiry or pre-implementation phase of an evaluation. However, both approaches have their own limitations—primarily the high intensity of resources and time required and the inability to make connections from determinants to outcomes. We combined implementation mapping and the IRLM with rapid qualitative analysis and clinical partner feedback to develop and test a seven-step approach for rapid contextual inquiry.

## Data Availability

De-identified data supporting the conclusions of this article will be made available by the authors upon reasonable request.

## Footnotes

^1^The CFIR-ERIC Matching Tool is available here: https://cfirguide.org/downloads/cfir-eric-matching-tool.
