# Supplementary material for: Rapid and pragmatic implementation mapping for pre-implementation contextual analysis in multisite implementation trials
Source: Front Health Serv. 2026 Jul 3;6:1782980. doi: 10.3389/frhs.2026.1782980 (PMC13375790; doi:10.3389/frhs.2026.1782980)
Supplement: Supplementary file 1 [file Table1.docx]

#

# Summary Table (Step 3)

| Domain | Determinant | Change Objective(s) |
| --- | --- | --- |
| Guideline Factors | | |
|  |  |  |
| Individual Health Professional Factors | | |
|  |  |  |
| Patient Factors | | |
|  |  |  |
| Professional Interactions | | |
|  |  |  |
| Incentives and Resources | | |
|  |  |  |
| Capacity for Organizational Change | | |
|  |  |  |
| Social, Political, and Legal | | |
|  |  |  |

# Identification of Implementation Strategies (Step 4)

*Best used in Excel similar spreadsheet.*

| Domain | Determinant | Change Objective(s) | Theme | Implementation Strategy 1 | Implementation Strategy 2 | Theme Suggestions | Strategy Suggestions |
| --- | --- | --- | --- | --- | --- | --- | --- |
|  |  |  |  |  |  | Adaptation | Alter allowance structures |
|  |  |  |  |  |  | Adoption | Education |
|  |  |  |  |  |  | Assessments | Mandate Change |
|  |  |  |  |  |  | Attendance | Modeling/Coaching |
|  |  |  |  |  |  | Benefits | Promote Adaptability |
|  |  |  |  |  |  | Clinician Roles | Resource Sharing |
|  |  |  |  |  |  | Cognitive Aids |  |
|  |  |  |  |  |  | Collaboration |  |
|  |  |  |  |  |  | Communication |  |
|  |  |  |  |  |  | Core Components |  |
|  |  |  |  |  |  | Evaluation |  |
|  |  |  |  |  |  | Financial |  |
|  |  |  |  |  |  | Lessons Learned |  |
|  |  |  |  |  |  | Outcomes |  |
|  |  |  |  |  |  | Patient Support |  |
|  |  |  |  |  |  | Policy |  |
|  |  |  |  |  |  | Relative Advantage |  |
|  |  |  |  |  |  | Relative Fit |  |
|  |  |  |  |  |  | Resources |  |
|  |  |  |  |  |  | Training |  |

# Completion of Implementation Research Logic Model (Step 5)

# Completion of tailored implementation blueprint (Step 7)

| Implementation Strategies | Actor | Action | Action Target | Temporality | Dose | Implementation outcome affected | Justification (APEASE Criteria) |
| --- | --- | --- | --- | --- | --- | --- | --- |
|  |  |  |  |  |  |  |  |
|  |  |  |  |  |  |  |  |
|  |  |  |  |  |  |  |  |
|  |  |  |  |  |  |  |  |
|  |  |  |  |  |  |  |  |
|  |  |  |  |  |  |  |  |
|  |  |  |  |  |  |  |  |
|  |  |  |  |  |  |  |  |
